# Real‐World Use of Normothermic Machine Perfusion in Liver Transplantation: A Multi‐Institutional Experience From the National Organ Perfusion Registry

**DOI:** 10.1111/ctr.70664

**Published:** 2026-08-31

**Authors:** Alexander R. Cortez, Wei San Loh, Sarah Sheskey, Hillary J. Braun, Aashna Mehta, Christopher Connelly, Austin Schenk, Musab Alebrahim, Raj Patel, Lori A. McLamb, Andrew Barbas, Steven A. Wisel, Garrett R. Roll, Kyle H. Sheetz

**Affiliations:** ^1^ Division of Transplant Surgery Mayo Clinic Rochester Minnesota USA; ^2^ Section of Transplant Surgery University of Michigan Ann Arbor Michigan USA; ^3^ Department of Surgery Division of Transplant University of California San Francisco San Francisco California USA; ^4^ Department of Surgery Oregon Health & Science University Portland Oregon USA; ^5^ Department of Surgery Ohio State University Wexner Medical Center Columbus Ohio USA; ^6^ Department of Surgery University of Arkansas for Medical Sciences Little Rock Arkansas USA; ^7^ Department of Surgery Duke University Hospital Durham North Carolina USA; ^8^ Comprehensive Transplant Center Cedars‐Sinai Medical Center Los Angeles California USA

**Keywords:** donation after cardiac death, liver transplantation, machine perfusion, normothermic perfusion

## Abstract

Normothermic machine perfusion (NMP) has enabled increased utilization of high‐risk liver allografts; however, real‐world data describing use, perfusion logistics, and associated outcomes remain limited. We established the National Organ Perfusion Registry (NOPR), a multicenter collaborative registry designed to capture granular perfusion data not available in the national transplant datasets. Adult liver transplant recipients who underwent transplantation using NMP between 2022 and 2024 at nine U.S. centers were included. Registry data were linked to the United Network for Organ Sharing (UNOS) Standard Transplant Analysis and Research files to obtain donor and recipient characteristics and post‐transplant outcomes. Descriptive analyzes were performed to characterize the indications for NMP use, procurement practices, perfusion timing parameters, and early graft outcomes. A total of 545 NMP liver transplants were analyzed, including 282 donations after circulatory death (DCD) and 263 donations after brain death (DBD). DCD was the most common indication for NMP, and its incidence increased substantially during the study period. Perfusion practices varied, including procurement strategy, device use, time from cross‐clamp to NMP initiation, and total perfusion duration. Despite this heterogeneity, early outcomes were favorable, with a 90‐day death‐censored graft failure rate of 1.7%, and no difference between DCD and DBD grafts. Recipients of DCD grafts had a shorter post‐transplant length of stay than DBD recipients. In this multicenter, real‐world cohort analysis, NMP was associated with low rates of early graft failure across diverse centers and perfusion practices. These findings support the feasibility of contemporary NMP use and underscore the importance of continued multicenter evaluation to refine perfusion strategies.

**Abbreviations**
ANOVA: Analysis of Variance
BMI: body mass index
CIT: cold ischemic time
DBD: Donation after brain death
DCD: Donation after circulatory death
EAD: early allograft dysfunction
HCC: hepatocellular carcinoma
IRB: Internal Review Board
IV: intravenous
MELD: Model for End‐Stage Liver Disease
NMP: Normothermic machine perfusion
NOP: National OCS Program
NOPR: National Organ Perfusion Registry
OCS: Organ Care System
OPTN: Organ Procurement & Transplant Network
PRS: post‐reperfusion syndrome
SCS: static cold storage
SD: standard deviations
SRTR: Scientific Registry of Transplant Recipients
STAR: Standard Transplant Analysis and Research
UNOS: United Network for Organ Sharing

## 1 Introduction

The need for suitable allografts for liver transplantation has prompted the development of strategies to expand the donor pool. This includes identifying mechanisms to reduce complication rates and improve utilization when transplanting livers from high‐risk donors, such as older donors, donors after circulatory death (DCD), and steatotic grafts. Normothermic machine perfusion (NMP) has emerged as a promising strategy to improve organ preservation and facilitate the use of high‐risk grafts. By minimizing cold ischemic time (CIT) and then maintaining the liver in a metabolically active state, NMP enables the restoration of cellular energy stores, mitigates ischemia‐reperfusion injury, and allows for functional assessment during preservation [1].

Early randomized trials demonstrated that NMP reduced the incidence of early allograft dysfunction (EAD) and post‐reperfusion syndrome PRS, [2, 3, 4] with one study reporting a statistically significant reduction in ischemic biliary complications [4]. This led to the commercial availability of NMP, and subsequently, there has been rapid clinical adoption. As centers increased their experience with NMP, several single‐center studies have demonstrated increased utilization with comparable or improved short‐ and long‐term outcomes compared to static cold storage (SCS) [5, 6, 7, 8]. More recently, an analysis using the United Network for Organ Sharing (UNOS)/Organ Procurement & Transplant Network (OPTN) database found that NMP has allowed for the expansion of transplants using organs from higher‐risk donors and reduction of non‐use with acceptable outcomes nationally [9].

As clinical experience with NMP has grown, attention has shifted from demonstrating its feasibility to refining its applications beyond the inclusion criteria from the original clinical trials. Clinically relevant considerations around NMP that are not well defined include the indication(s) for use and the temporal logistics of perfusion (including the time from cross‐clamp to initiation of NMP and the total NMP duration). Unfortunately, national databases such as the Scientific Registry of Transplant Recipients (SRTR) and UNOS/OPTN do not capture perfusion parameters, and single‐center studies are limited in their sample sizes. Moreover, the lack of granularity in large‐scale perfusion studies does not provide real‐world data on large‐scale NMP use.

To address these methodological limitations and further study the use and impact of NMP, we established the National Organ Perfusion Registry (NOPR). This study represents preliminary work from the NOPR and has three aims. First, to determine the feasibility and effectiveness of data linkage with existing data sources to facilitate multicenter evaluation of perfusion technologies on liver transplant outcomes. Second, to provide an exploratory analysis of NMP perfusion data that generates hypotheses for future study. Finally, to characterize real‐world perfusion practices and descriptively explore associations between perfusion logistics and post‐transplant outcomes.

## 2 Methods

### 2.1 Data Source and Data Collection

The NOPR is a consortium of nine transplant centers that has collected data on liver transplants using NMP since 2022. Using REDcap, each program enters NMP cases at their center. The registry collects center name, UNOS ID, Match ID, NMP device (TransMedics Organ Care System [OCS]), OrganOx metra [Terumo, Madison, New Jersey, USA], or other), and if the liver was successfully transplanted into a recipient. If transplanted, times are entered for donor cross‐clamp, NMP start time, NMP end time, and liver reperfusion time. Procurement team is reported as using the accepting center's surgeons or an external procurement service. For the OCS device, TransMedics offers a “full service” National OCS Program (NOP) that can be requested by the accepting center. In all cases, the accepting center dictates all procurement and acceptance decisions including visual assessment, need for biopsy, and placing the organ on pump. Reasons for NMP included donor‐related factors (DCD, steatosis, age, pre‐recovery biopsy not available, or other), recipient‐related factors (previous surgery, liver/kidney transplant, liver/heart transplant, portal vein thrombosis, pulmonary hypertension, and severe portal hypertension), and logistic factors (prolonged CIT expected, operating room availability, extra time necessary to prepare recipient, and late reallocation to recipient). Not all livers had a secondary indication. The indication(s) for NMP utilization were determined by the accepting surgeon at the time of organ offer and upon sending of the procurement team. Any vascular injury that occurred during procurement or the need for arterial reconstruction prior to initiating perfusion was also recorded.

The cohort for this study includes adult recipients who underwent liver transplantation between 2022 and 2024. To obtain donor and recipient characteristics and outcomes, the NOPR registry was linked to UNOS Standard Transplant Analysis and Research (STAR) files using unique donor identifiers. Data collected included donor demographics, comorbidities, cause of death, donation type (DCD vs. donation after brain death [DBD]), recipient clinical status (e.g., Model for End‐Stage Liver Disease [MELD] score, hepatocellular carcinoma [HCC] diagnosis), and transplant outcomes. Recipient MELD (at listing and at time of transplant) are medical/calculated scores and are not adjusted for exception points. HCC diagnosis was reported separately as a recipient characteristic in the STAR file.

Post‐transplant outcomes include 90‐day and one‐year mortality and death‐censored graft failure. Mortality was defined as death within 90 days or 365 days after transplant, respectively. Death‐censored graft failure was defined as graft failure within the corresponding time window, excluding deaths with a functioning graft. The STAR file used for analysis was updated in 2025, and all patients in the cohort had at least one year of follow up. A sensitivity analysis was performed excluding combined multiorgan liver transplants (*N =* 10 liver/kidney and *N =* 4 liver/heart) to evaluate outcomes among liver‐only transplant recipients.

### 2.2 Statistical Analysis

Descriptive statistical methods were used to summarize donor, graft, perfusion, and recipient characteristics. Continuous variables were summarized using means and standard deviations (SD), and categorical variables were summarized using counts and percentages. Group comparisons between recipients with DCD versus DBD donor status and across NMP device types were conducted using analysis of variance (ANOVA) for continuous variables or Pearson's chi‐square test for categorical variables, as appropriate. Exploratory adjusted analysis of post‐transplant LOS between DBD and DCD recipients was performed using a generalized linear model with robust standard errors to account for differences in recipient MELD score and dialysis at the time of transplant and evaluated using Wald test.

Normothermic machine perfusion (NMP) time intervals, including NMP initiation (time from cross‐clamp to NMP initiation) and total NMP duration, were evaluated descriptively and compared across device types and procurement teams. Statistical significance was set at *p <*0.05 (2‐tailed). All statistical analyzes were performed using Stata (version 18, StatsCorp LLC, College Station, TX, USA) between July and November 2025. This study was approved by the University of Michigan Internal Review Board (IRB) and by each participating center as required and was deemed exempt.

## 3 Results

### 3.1 NOPR *Study Cohort*

A total of 545 liver transplants utilizing NMP at nine centers were included in the study. There were 263 (48.3%) DBD grafts and 282 (51.7%) DCD grafts. Overall, the average donor was 47 ± 13.8 years old, male (59.8%), with a body mass index (BMI) of 30 ± 8.0. The average recipient was 56.6 ± 10.9 years old, male (63.7%), with a BMI of 29.7 ± 6.4. The average MELD at time of transplant was 22.1 ± 8.9, 25% of recipients had HCC, and 13.1% were on dialysis at the time of transplant. The donor and recipient characteristics for the overall cohort and the DBD and DCD subgroups are reported in Table 1.

**TABLE 1:** Cohort demographics and outcomes.

	All (*N =* 545)	DBD (*N =* 263)	DCD (*N =* 282)	*p*
**Donor Characteristics**				
DCD status, n (%)	282 (51.7%)			
Age, mean (SD), y	47.0 (13.8)	48.0 (14.8)	46.1 (12.8)	0.11
BMI, mean (SD)	30.0 (8.0)	30.9 (8.8)	29.2 (7.2)	0.013
Female, n (%)	219 (40.2%)	112 (42.6%)	107 (37.9%)	0.27
Cause of Death, n (%)				
*Anoxia*	298 (54.7%)	143 (54.4%)	155 (55.0%)	0.89
*Cerebrovascular / Stroke*	137 (25.1%)	76 (28.9%)	61 (21.6%)	0.051
*Head Trauma*	94 (17.2%)	38 (14.4%)	56 (19.9%)	0.095
*Other*	16 (2.9%)	6 (2.3%)	10 (3.5%)	0.38
Had Liver Biopsy, n (%)	285 (54.3%)	165 (65.7%)	120 (43.8%)	<0.001
Diabetes, n (%)	87 (16.4%)	46 (18.3%)	41 (14.7%)	0.27
Hypertension, n (%)	233 (43.7%)	121 (47.6%)	112 (40.1%)	0.081
Heavy alcohol use, n (%)	115 (22.3%)	49 (20.0%)	66 (24.4%)	0.24
Cancer, n (%)	31 (5.8%)	17 (6.7%)	14 (5.0%)	0.42
I.V. Drug Usage, n (%)	74 (14.1%)	39 (15.6%)	35 (12.7%)	0.34
**Recipient Characteristics**				
Age, mean (SD), y	56.6 (10.9)	55.9 (11.8)	57.2 (10.1)	0.18
BMI, mean (SD)	29.7 (6.4)	29.4 (6.3)	30.0 (6.5)	0.24
Female, n (%)	198 (36.3%)	94 (35.7%)	104 (36.9%)	0.78
MELD score at listing, mean (SD)	20.1 (8.9)	21.5 (9.8)	18.8 (7.9)	<0.001
MELD score at transplant, mean (SD)	22.1 (8.9)	23.9 (9.5)	20.4 (8.0)	<0.001
Total days on waiting list, mean (SD)	223.3 (433.2)	247.8 (511.1)	200.4 (344.5)	0.20
HCC Diagnosis, n (%)	129 (25.0%)	65 (26.5%)	64 (23.7%)	0.46
Prior liver transplant, n (%)	26 (4.8%)	20 (7.6%)	6 (2.1%)	0.003
Dialysis prior week to transplant, n (%)	71 (13.1%)	49 (18.6%)	22 (7.8%)	<0.001
90‐day graft failure, n (%)	9 (1.7%)	5 (1.9%)	4 (1.4%)	0.66
1‐year graft failure, n (%)	14 (2.6%)	7 (2.7%)	7 (2.5%)	0.89
90‐day mortality, n (%)	11 (2.0%)	5 (1.9%)	6 (2.1%)	0.85
1‐year mortality, n (%)	25 (4.6%)	14 (5.3%)	11 (3.9%)	0.43
Length of stay post‐transplant hospitalization, average days (SD)	13.3 (13.5)	15.5 (15.1)	11.4 (11.6)	<0.001
Retransplantation, n (%)	13 (2.4%)	7 (2.7%)	6 (2.1%)	0.68

Abbreviations: BMI, body mass index; DBD, donation after brain death; DCD, donation after circulatory death; HCC, hepatocellular carcinoma; I.V., intravenous; MELD, Model for End‐Stage Liver Disease; SD, standard deviation,.

The post‐transplant outcomes are also listed in Table 1. In the overall cohort, the 90‐day graft failure rate was 1.7% (*n =* 9/545), and the 90‐day mortality rate was 2%. The mean LOS for DBD recipients was longer compared to DCD recipients (15.5 ± 15.1 vs 11.4 ± 11.6 days, *p <*0.001). There were no differences in the 90‐day and one‐year graft failures between the groups. After adjusting for MELD score and dialysis at the time of transplant, DCD recipients continued to demonstrate shorter mean LOS compared to DBD recipients (12.1 vs 14.5 days, *p =* 0.033). Similar results were seen in a sensitivity analysis among liver‐only transplants after excluding the cohort of multiorgan liver transplants (*N =* 10 liver/kidney and *N =* 4 liver/heart) (Table S1).

### 3.2 Indications for NMP

The indications for NMP are listed in Table 2. The most common donor‐related reasons were DCD (*n =* 282, 51.8%), steatosis (*n =* 61, 11.2%), and age (*n =* 48, 8.8%). Recipient‐ and logistic‐related reasons were less common overall. Other common reasons included prior surgery (*n =* 34, 6.2%) and expected prolonged CIT (*n =* 37, 6.8%). The indication patterns shift over time, as shown in Table 2. Overall, NMP utilization increased from 68 cases in 2022 to 206 in 2023 and 269 in 2024, with DCD indications rising both in absolute count (27 to 152) and proportion of cases (39.7% to 56.5%). Several non‐donor indications also increased over time, including anticipated prolonged CIT (3 to 21) and prior surgery (6 to 18), while steatosis as the primary indication declined proportionally from 26.5% in 2022 to 7.4% in 2024. Indications also varied by center, with evidence of center‐specific clustering by primary indication for NMP. As reported in Table S2, among 282 DCD livers, 238 (84.4%) had a secondary indication. The most common were prolonged CIT expected (*n =* 42, 14.9%), donor age (*n =* 43, 15.2%), and graft steatosis (*n =* 30, 10.6%).

**TABLE 2:** Distribution of primary indications for NMP use (*N =* 545).

	Primary Indication for NMP	Transplant Year			N (%)
		2022	2023	2024	
**Donor**	Donation after cardiac death (DCD)	27	103	152	282 (51.8%)
	Graft steatosis	18	23	20	61 (11.2%)
	Donor age	7	17	24	48 (8.8%)
	Marginal donor (other than above)	3	12	8	23 (4.2%)
	Pre‐recovery biopsy not available	0	1	0	1 (0.2%)
**Recipient**	Previous surgery	6	10	18	34 (6.2%)
	Liver/kidney transplant	0	2	8	10 (1.8%)
	Liver/heart transplant	0	3	1	4 (0.7%)
	Portal vein thrombosis	0	3	4	7 (1.3%)
	Pulmonary hypertension	0	3	3	6 (1.1%)
	Severe portal hypertension	1	0	0	1 (0.2%)
**Other**	Prolonged cold ischemia expected	3	13	21	37 (6.8%)
	Operating room availability	1	9	3	13 (2.4%)
	Extra time necessary to prepare recipient	1	5	4	10 (1.8%)
	Late reallocation to recipient	1	2	3	6 (1.1%)
	Missing values^a^	0	2	0	2 (0.4%)

^a^ Two cases had missing primary indication data due to incomplete registry reporting from a single participating center.

### 3.3 NMP Perfusion Metrics

Of the 545 livers that underwent NMP and were transplanted, 83 (15.2%) were perfused on OrganOx and 462 (84.8%) were perfused on TransMedics. The mean NMP duration was 12.6 ± 5.1 h, with a significant difference between devices (OrganOx: 11.5 ± 3.8 vs. TransMedics: 12.8 ± 5.3 h, *p =* 0.033). The NMP initiation was significantly longer in the OrganOx group (265.3 ± 125.4 vs 142.5 ± 61.2 min, *p <*0.001). The subsequent implantation time (from the end of NMP to liver reperfusion) was similar between the groups (60.4 ± 22.9 vs 66.6 ± 27.9, *p =* 0.058) (Table 3). About half of the livers (270 of 540) were recovered by the recipient center's procuring surgeons, while the remaining livers were recovered via local recovery or a national procurement service. The use of procurement services was more common with TransMedics than with OrganOx (54.1% vs. 24.1%, *p <*0.001). Arterial reconstruction prior to perfusion was required in 99 cases (18.2%), and vascular injuries occurred in 10 cases (1.8%). There was no difference in injuries between procurements performed by the recipient team and local recovery/national procurement services (1.1% vs. 2.6%, *p =* 0.20). There was also no difference in time to initiation of NMP between procurements performed by the recipient team and a local recovery/national procurement service (166.6 ± 88.9 vs 155.0 ± 83.9, *p =* 0.12).

**TABLE 3:** Perfusion times and procurement by NMP device (OrganOx metra vs. TransMedics OCS).

	All Devices (*N =* 545)	OrganOx metra (*N =* 83)	TransMedics OCS (*N =* 462)	*p*
**NMP duration, mean (SD), hours**	12.6 (5.1)	11.5 (3.8)	12.8 (5.3)	0.033
**NMP initiation, mean (SD), minutes**	161.2 (86.6)	265.3 (125.4)	142.5 (61.2)	< 0.001
**NMP to Liver Reperfusion, mean (SD), minutes**	65.6 (27.3)	60.4 (22.9)	66.6 (27.9)	0.058
**Procurement Service (%)**				<0.001
Recipient center's normothermic perfusion trained team	270 (49.5)	59 (71.1)	211 (45.7)	
National normothermic perfusion team	270 (49.5)	20 (24.1)	250 (54.1)	
Missing values^a^	5 (1.0)	4 (4.8)	1 (0.2)	
**Need for arterial reconstruction prior to initiating perfusion (%)**	99 (18.2)	14 (16.9)	85 (18.4)	0.74
**Had vascular injury (%)**	10 (1.8%)	3 (3.6%)	7 (1.5%)	0.19

Abbreviations: NMP, normothermic machine perfusion; OCS, organ care system; SD, standard deviation.

^a^ Procurement service information was unavailable for five cases due to incomplete registry reporting.

### 3.4 Post‐Transplant Outcomes

Ninety‐day graft failure occurred in nine recipients (1.7%) among the cohort (Table 4). Four of the nine graft failures (44.4%) were DCD livers. Numerically, donor and recipient age, BMI and MELD were similar between functioning grafts and those that failed. Of the nine graft failures, none had vascular injury, eight (88.9%) were perfused on TransMedics, and six (67%) were organs procured by a national normothermic perfusion team. There was no statistically significant difference in the likelihood of graft loss based on the NMP device. The average NMP initiation time was 135.3 ± 36.2 min, and NMP duration was 15.4 h ± 6.1 h for recipients with early graft failure. Numerically, these were similar to the overall cohort but limited statistical comparison given sample size.

**TABLE 4:** Descriptive statistics for graft survived vs failed within 90‐days post‐transplant.

	Total *N =* 545	Non‐failure *N =* 536	Graft Failure *N =* 9	p
**Procurement Characteristics** Vascular injury, n (%)	10 (1.8%)	10 (1.9%)	0	0.68
Procurement Service, n (%)				0.31
Recipient center's trained team	270 (49.5%)	267 (49.8%)	3 (33.3%)	
National team	270 (49.5%)	264 (49.3%)	6 (66.7%)	
Missing values	5 (1.0%)	5 (0.9%)		
Normothermic perfusion device, n (%)				0.73
OrganOx metra	83 (15.2%)	82 (15.3%)	1 (11.1%)	
TransMedics Organ Care System (OCS)	462 (84.8%)	454 (84.7%)	8 (88.9%)	
NMP duration, mean (SD), hours	12.6 (5.1)	12.6 (5.1)	15.4 (6.1)	0.1
NMP initiation, mean (SD), minutes	161.2 (86.6)	161.6 (87.1)	135.3 (36.2)	0.37
NMP to Liver Reperfusion, mean (SD), minutes	65.6 (27.3)	65.5 (27.3)	75.0 (23.8)	0.3
**Donor Characteristics**				
DCD status, n (%)	282 (51.7%)	278 (51.9%)	4 (44.4%)	0.66
Age, mean (SD), y	47.0 (13.8)	47.0 (13.9)	51.4 (9.3)	0.34
BMI, mean (SD)	30.0 (8.0)	30.0 (8.0)	29.8 (7.6)	0.94
**Recipient Characteristics**				
Age, mean (SD), y	56.6 (10.9)	56.5 (11.0)	58.6 (7.3)	0.58
BMI, mean (SD)	29.7 (6.4)	29.7 (6.4)	31.1 (5.2)	0.52
MELD score at listing, mean (SD)	20.1 (8.9)	20.1 (9.0)	20.4 (6.8)	0.9
MELD score at transplant, mean (SD)	22.1 (8.9)	22.1 (9.0)	21.6 (7.2)	0.86
Total days on waiting list, mean (SD)	223.3 (433.2)	222.9 (436.1)	246.0 (213.7)	0.87
Had prior liver transplant, n (%)	26 (4.8%)	25 (4.7%)	1 (11.1%)	0.37
Had dialysis prior week to transplant, n (%)	71 (13.1%)	70 (13.1%)	1 (11.1%)	0.86
Length of stay post‐transplant hospitalization, average days (SD)	13.3 (13.5)	13.3 (13.4)	17.2 (18.1)	0.52

BMI, body mass index, DCD, donation after circulatory death, MELD, Model for End‐Stage Liver Disease, NMP, normothermic machine perfusion, SD, standard deviation.

The nine graft failures were also compared by donor type (DBD vs. DCD) (Table S3). Ninety‐day graft failure was 1.9% (n = 5) among DBD grafts and 1.4% (*n =* 4) among DCD grafts (*p =* 0.66). Neither group had a vascular injury at the time of procurement. The NMP initiation was similar between groups (130.6 ± 30.6 vs 141.2 ± 46.5 min, *p =* 0.69), but total NMP duration was longer in the DCD group than DBD group (20.5 ± 3.8 vs 11.3 ± 4.1, *p =* 0.011). There were some numeric differences in donor characteristics, but limited statistical comparisons given the small overall cohort sizes.

**FIGURE 1:**
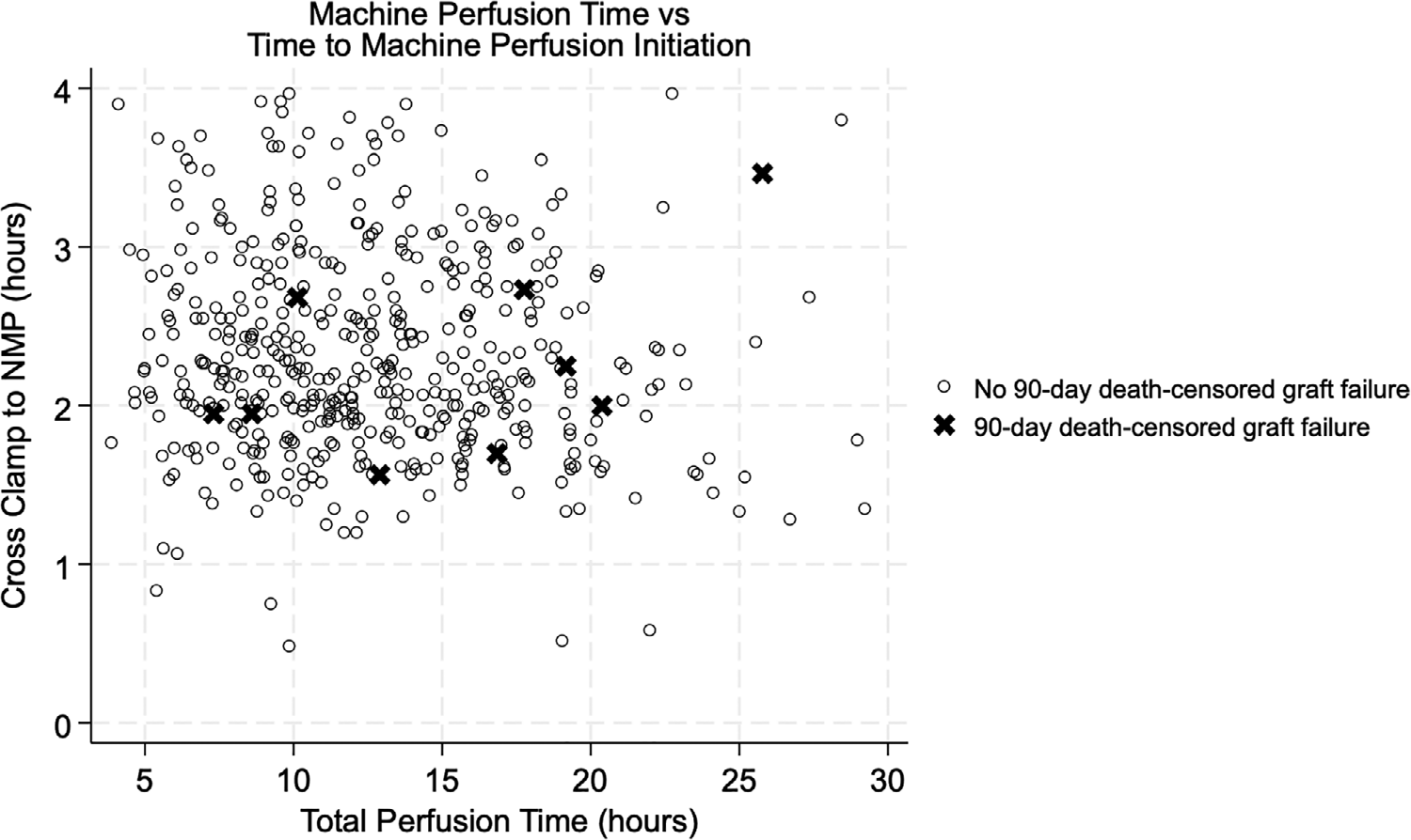
90‐day death‐censored graft outcomes stratified by NMP initiation and NMP duration.

Finally, graft failure as a function of the interaction between NMP initiation and NMP duration was graphically examined, as shown in Figure 1.

## 4 Discussion

In this multicenter study, we describe the real‐world use of NMP across nine U.S. liver transplant centers. Early graft outcomes, including 90‐day graft failure and LOS, were examined descriptively across a range of perfusion practices; however, event rates were low, limiting definitive conclusions. By linking multicenter registry data to the UNOS STAR file, this study demonstrates the feasibility of capturing detailed perfusion logistics with standardized post‐transplant outcomes. However, the lack of granularity in reporting of certain post‐transplant outcomes and low event rates introduced limitations into this research approach. Finally, this study is the first to systematically characterize perfusion times in liver transplantation, which is not available in existing national datasets. Together, these data provide a multicenter view of contemporary indications, logistics, and perfusion practices associated with NMP use in liver transplantation.

Clinical adoption of NMP has expanded rapidly since commercial availability, with national data demonstrating a substantial increase in the proportion of transplanted allografts preserved using NMP [9]. In our multicenter cohort, the most common indication for NMP was DCD, which was the primary indication for use in over 50% of cases. The predominance of DCD grafts reflects broader national trends toward increased utilization of DCD livers. Historically, DCD liver transplantation was concentrated among a limited number of high‐volume centers, [10] but more recent national analyzes demonstrate increased DCD utilization at small, medium, and large programs, and a declining concentration of DCD volume among the highest‐volume centers [11]. This has occurred alongside increased use of NMP, which may suggest that machine perfusion lowers the clinical or logistical barriers to DCD use. The clinical importance of these practices is underscored by the fact that acceptance of DCD liver offers is associated with improved waitlist outcomes [12, 13, 14]. In our cohort, DCD livers were transplanted into recipients with numerically shorter wait times at a lower MELD and had shorter LOS compared to DBD livers. This was replicated in our adjusted analysis accounting for recipient comorbidities, though residual confounding may limit the ability to further understand this impact of NMP.

Historically, DCD grafts were procured from ideal donors without additional risk factors and used for straightforward transplants with short CIT; however, our data found that over 70% of DCD grafts had additional risk factors (donor age, steatosis, prolonged CIT, simultaneous organ transplant), and these secondary indications increased in frequency over the study period. Collectively, this suggests that as centers gained experience with DCD organ use, they were willing to take on additional donor risk factors, as well as use these allografts for more complex transplant scenarios. Interestingly, we found steatosis as a primary indication for NMP decreased over the study, which may reflect early enthusiasm that was later tempered as centers experienced ongoing challenges associated with steatotic livers despite NMP. The higher biopsy rate observed among DBD donors in this study may reflect greater use of NMP for marginal DBD grafts with visual features prompting histologic assessment, whereas higher‐risk DCD grafts may have been excluded earlier in the donor evaluation process. We speculate that this finding reflects differences in donor selection practices; however, this hypothesis cannot be evaluated with the present dataset and warrants prospective evaluation.

In addition to characterizing the indications for NMP utilization, this study highlights the substantial heterogeneity in NMP logistics across centers. Approximately half of the allografts were recovered by the recipient center's team, while the remainder were recovered using external procurement services. Livers preserved on TransMedics were more commonly recovered by an external procurement team compared to livers preserved on OrganOx (54% vs. 25%, *p <*0.001), consistent with the former offering a national procurement “full service” recovery option. OrganOx livers had a longer time to initiation of NMP (265 vs. 143 min, *p <*0.01), likely reflecting its more common use in a back‐to‐base fashion. These observations are consistent with a national survey by Otajonova et al., which demonstrated variability in DCD liver graft acceptance based on procuring surgeon, procurement technique, and storage modality [15]. These differences should not be interpreted as evidence of intrinsic device‐related effects but rather differing procurement workflows and utilization patterns that cannot be studied in the present analysis. Notably, two‐thirds of early graft failures occurred among organs recovered by a national procurement team, but no inferential conclusions should be drawn regarding inferior outcomes associated with external procurement teams given the small event rate. Taken together, these findings in procurement heterogeneity highlight procurement workflow as an important and understudied aspect of contemporary NMP practice that should be prioritized in future studies.

Despite variability in procurement personnel vascular injury during recovery was uncommon (<2%) and did not differ when recovered by the recipient team vs an external procurement service. However, nearly 20% of grafts required arterial reconstruction prior to the initiation of perfusion, underscoring the technical complexity inherent to NMP recoveries. These findings highlight the increasing technical demands of procurement surgery and highlight the implications for training among transplant fellows and procurement surgeons. Prior studies have demonstrated substantial variability in procurement training exposure among transplant trainees, including limited formal didactic instruction and variable experience with DCD recovery, while structured training interventions have been shown to improve confidence and competence in donor recovery tasks [16, 17]. In addition, clinical perspectives describe the personnel and procedural demands associated with NMP, including cannulation, graft preparation, and coordination under time constraints, further emphasizing the complexity of contemporary procurement practice [18].

As NMP has been widely adopted into clinical practice, the timing of perfusion initiation and total perfusion duration have emerged as important practical considerations. Early NMP randomized trials were conducted under relatively standardized conditions with narrow ranges of CIT and relatively short perfusion durations (approximately 4–6 h) and were not designed to study the clinical impact of these perfusion parameters [3, 4]. Our multicenter cohort demonstrates the real‐world experience of longer and more variable perfusion durations and time to initiation of NMP. In this context, we examined 90‐day graft failure and found it to be uncommon, providing descriptive evidence that variability in perfusion logistics can occur without frequent early graft loss. However, further work is needed to establish the impact of time to NMP initiation or total perfusion duration and is limited by a lack of perfusion data in any large series. Despite excellent early graft survival, very high‐risk allografts in the VITTAL trial, which averaged 9 h CIT, demonstrated high rates of biliary complications suggesting there are likely upper limits of CIT tolerance prior to NMP initiation [19]. Similarly, EAD risk was associated with prolonged time to initiation of NMP in DCD livers [20]. Though our event rate of graft failure was low, we did not see any signal associated with either of these perfusion variables. As graft failure rates continue to decline and the impact of EAD is reduced, reliance on these traditional metrics may inadequately capture meaningful differences in contemporary transplant outcome and inclusion of reliable perioperative variables within national datasets is essential to facilitate future machine perfusion research.

This study has several limitations that should be acknowledged when interpreting these data. First, although this represents one of the largest multicenter cohorts with detailed perfusion timing data, the overall incidence of early graft failure was low, limiting the statistical power and precluding more granular risk stratification or causal inference. Second, as an observational study conducted at centers that represent early adopters of NMP, the findings may not be fully generalizable to all transplant programs. Additionally, robust matching and adjustment for potential confounders were limited by sample size and cohort heterogeneity. Third, viability assessment and perfusion parameters were not captured, limiting mechanistic insight into observed outcome differences. In addition, analyzes comparing perfusion devices are limited by cohort heterogeneity and the exploratory nature of the analysis. However, these comparisons remain important given the technical and logistical differences between platforms, including perfusion characteristics, at‐source versus back‐to‐base workflows, and differences in procurement and cannulation practices. Fourth, linkage to the UNOS STAR file limited outcome granularity beyond LOS and graft failure, precluding a detailed assessment of other clinically relevant postoperative outcomes, such as early allograft dysfunction, post‐reperfusion syndrome, vascular complications, biliary complications, and composite morbidity measures.

## 5 Conclusion

In this multicenter, real‐world study of liver transplantation using NMP, we observed low rates of early graft failure with no significant difference between DCD and DBD allografts or across variable perfusion initiation and duration practices. These findings demonstrate that in the NMP era, DCD liver transplantation in appropriately selected recipients may achieve early graft outcomes comparable to those of DBD transplantation across diverse transplant centers. This study also provides novel multicenter data characterizing real‐world indications for NMP utilization, contemporary procurement and perfusion logistics, and substantial variability in perfusion initiation and duration practices across transplant centers. While data linkage to existing datasets demonstrates feasibility in streamlining multicenter data, the lack of quality reporting and granularity in existing transplant datasets represents an important opportunity area to improve future research. These results support the expanding role of NMP and underscore the importance of continued multicenter evaluations to refine perfusion strategies and optimize outcomes.

## Author Contributions

**Alexander R. Cortez, Steven A. Wisel, Garrett R. Roll, Kyle H. Sheetz:** study concept and design, data interpretation, drafting article and critical revision of article. **Wei San Loh, Sarah Sheskey:** data collection, data analysis, statistical support, critical review and approval of the article. **Hillary J. Braun, Aashna Mehta, Christopher Connelly, Austin Schenk, Musab Alebrahim, Raj Patel, Lori A. McLamb, Andrew Barbas**: data collection, data interpretation, critical revision and approval of article.

## Funding

This work was supported by ASTS Ronald and JoAnn Busuttil Surgeon Scientist.

## Conflicts of Interest

The authors declare no conflicts of interest.

## Supporting information




**Supporting Information:** ctr70664‐sup‐0001‐Tables.docx

## Data Availability

The data that support the findings of this study are available on request from the corresponding author. The data are not publicly available due to privacy or ethical restrictions.
