# Supplementary material for: Real‐World Use of Normothermic Machine Perfusion in Liver Transplantation: A Multi‐Institutional Experience From the National Organ Perfusion Registry
Source: Clin Transplant. 2026 Aug 31;40(9):e70664. doi: 10.1111/ctr.70664 (PMC13528972; doi:10.1111/ctr.70664)
Supplement: Supplementary file 1 [file CTR-40-e70664-s001.docx]

**Supplemental Table 1. Outcomes among subgroup of liver-only transplants**

| **Recipient Post-Transplant Outcome** | **Total** | | **DBD** | | **DCD** | | **p-value** | |
| --- | --- | --- | --- | --- | --- | --- | --- | --- |
|  | N=531 | N=250 | | N=281 | |  | |  |
| 90-day graft failure, n (%) | 9 (1.7%) | 5 (2.0%) | | 4 (1.4%) | | 0.61 | |  |
| 1-year graft failure, n (%) | 14 (2.6%) | 7 (2.8%) | | 7 (2.5%) | | 0.82 | |  |
| 90-day mortality, n (%) | 11 (2.1%) | 5 (2.0%) | | 6 (2.1%) | | 0.91 | |  |
| 1-year mortality, n (%) | 25 (4.7%) | 14 (5.6%) | | 11 (3.9%) | | 0.36 | |  |
| Length of stay post-transplant hospitalization, average days (SD) | 13.0 (13.1) | 15.0 (14.5) | | 11.3 (11.5) | | 0.002 | |  |
| Retransplantation, n (%) | 13 (2.4%) | 7 (2.8%) | | 6 (2.1%) | | 0.62 | |  |

*Excludes combined multiorgan liver transplants (N=10 liver/kidney and N=4 liver/heart)*

**Supplemental Table 2: Distribution of secondary indications among DCD NMP allografts (N=282)**

|  | **Secondary Indication for NMP** | **Transplant Year** | | | **N (%)** |  |
| --- | --- | --- | --- | --- | --- | --- |
|  |  | **2022** | **2023** | **2024** |  |  |
| **Donor** | Graft steatosis | 6 | 7 | 17 | 30 (10.6%) | |
|  | Donor age | 1 | 12 | 30 | 43 (15.2%) | |
|  | Marginal donor (other than above) | 0 | 11 | 11 | 22 (7.8%) | |
|  | Pre-recovery biopsy not available | 3 | 2 | 2 | 7 (2.5%) | |
|  |  |  |  |  |  | |
|  | Previous surgery | 3 | 11 | 16 | 30 (10.6%) | |
| **Recipient** | Liver/kidney transplant | 0 | 3 | 10 | 13 (4.6%) | |
|  | Liver/heart transplant | 0 | 0 | 0 | 0 | |
|  | Portal vein thrombosis | 1 | 7 | 12 | 20 (7.1%) | |
|  | Pulmonary hypertension | 0 | 0 | 0 | 0 | |
|  | Severe portal hypertension | 1 | 0 | 4 | 5 (1.8%) | |
|  |  |  |  |  |  | |
|  | Prolonged cold ischemia expected | 0 | 20 | 22 | 42 (14.9%) | |
| **Other** | Operating room availability | 1 | 2 | 4 | 7 (2.5%) | |
|  | Extra time necessary to prepare recipient | 3 | 3 | 5 | 11 (3.9%) | |
|  | Late reallocation to recipient | 1 | 3 | 4 | 8 (2.8%) | |
|  | No secondary indication |  |  |  | 44 (15.6%) | |

**Supplemental Table 3. Descriptive statistics for death-censored graft failure cases within 90-days post-transplant**

|  | **DBD**  N=5 | **DCD**  N=4 | **p** |
| --- | --- | --- | --- |
| **Procurement Characteristics** |  |  |  |
| Vascular injury, n (%) | 0 | 0 |  |
| Procurement Service, n (%) |  |  | 0.018 |
| - Recipient center's trained team | 0 (0%) | 3 (75%) |  |
| - National team | 5 (100%) | 1 (25%) |  |
| Normothermic perfusion device, n (%) |  |  | 0.24 |
| - OrganOx® metra® | 0 (0%) | 1 (25%) |  |
| - TransMedics® Organ Care System™ (OCS) | 5 (100%) | 3 (75%) |  |
| NMP duration, mean (SD), hours | 11.3 (4.1) | 20.5 (3.8) | 0.011 |
| NMP initiation, mean (SD), minutes | 130.6 (30.6) | 141.2 (46.5) | 0.69 |
| NMP to Liver Reperfusion, mean (SD), minutes | 84.4 (28.7) | 63.2 (9.3) | 0.20 |
| **Donor Characteristics** |  |  |  |
| Age, mean (SD), y | 47.6 (8.9) | 56.2 (8.4) | 0.18 |
| BMI, mean (SD) | 27.4 (7.0) | 32.8 (8.1) | 0.31 |
| **Recipient Characteristics** |  |  |  |
| Age, mean (SD), y | 58.0 (7.5) | 59.2 (8.1) | 0.82 |
| BMI, mean (SD) | 31.9 (4.5) | 30.1 (6.6) | 0.64 |
| MELD score at listing, mean (SD) | 22.2 (6.8) | 18.2 (7.1) | 0.42 |
| MELD score at transplant, mean (SD) | 21.8 (9.7) | 21.2 (3.4) | 0.92 |
| Total days on waiting list, mean (SD) | 263.6 (259.8) | 224.0 (175.1) | 0.80 |
| Had previous liver transplant, n (%) | 0 (0%) | 1 (25%) | 0.24 |
| Had dialysis prior week to transplant, n (%) | 1 (20%) | 0 (0%) | 0.34 |
| Length of stay post-transplant hospitalization, average days (SD) | 24.7 (21.1) | 6.0 (0.0) | 0.32 |
|  |  |  |  |

*BMI – body mass index, DBD – donation after brain death, DCD – donation after circulatory death, MELD – Model for End-Stage Liver Disease, SD – standard deviation,*
